# Surviving an out-of-hospital hypothermic cardiac arrest in the United Kingdom

**DOI:** 10.29045/14784726.2023.3.7.4.46

**Published:** 2023-03-01

**Authors:** Stuart Evans

**Affiliations:** West Midlands Ambulance Service University NHS Foundation Trust

**Keywords:** cardiac arrest, extracorporeal life support (ECLS), hypothermia, resuscitation

## Abstract

**Introduction:**

Hypothermia is an uncommon cause of cardiac arrest in the United Kingdom, and more commonly occurs in countries experiencing avalanches and significant winter climates; however, this case demonstrates that the presentation *can* occur in the United Kingdom. This case adds to a body of evidence that prolonged resuscitation can be successful in patients suffering a cardiac arrest secondary to hypothermia, leading to a good neurological outcome.

**Case presentation:**

The patient suffered a witnessed out-of-hospital cardiac arrest following rescue from a free-flowing river, and underwent prolonged resuscitation. The patient presented in persistent ventricular fibrillation, unresponsive to defibrillation attempts. An oesophageal probe recorded the patient’s temperature as 24°C. Rescuers were guided by the Resuscitation Council UK advanced life support algorithm to withhold drug therapy and limit defibrillation attempts to three, until the patient had been rewarmed to above 30°C. Appropriate triage of the patient to an extracorporeal life support (ECLS) capable centre allowed specialised treatment to be initiated, and culminated in successful resuscitation once normothermia was restored. After a short stay in intensive care, the patient was discharged for rehabilitation due to a hypoxic spinal cord injury before discharge home.

**Conclusion:**

This case highlights that hypothermia is a reversible cause of cardiac arrest, which needs to be recognised and acted upon appropriately to provide the best possible chance for a positive outcome. Low-reading thermometers capable of identifying the temperature thresholds stated in the Resuscitation Council UK guidelines are required, to allow clinicians to adapt their practice according to the presenting situation. Tympanic thermometers are often limited to their lowest recordable temperature, and invasive monitoring such as oesophageal or rectal probes are not common in UK ambulance service practice. With the necessary equipment, patients can be triaged to an ECLS-capable centre, allowing them to receive the specialist rewarming that they require.

## Introduction

NHS ambulance services attempt out-of-hospital resuscitation in approximately 30,000 people each year, with most cases occurring in the home or workplace (Resuscitation Council UK, 2021a). Of the cases where resuscitation is attempted, 9% of people survive to hospital discharge following out-of-hospital cardiac arrest.

UK life-support guidelines encourage clinicians to approach patients in a systematic manner and address any potential reversible cause for the cardiac arrest (Resuscitation Council UK, 2021b). Reversible causes are commonly taught using the ‘Four Hs and Four Ts’ approach (hypoxia, hypovolaemia, hypothermia, hyper-/hypo-kalaemia/metabolic, tension pneumothorax, cardiac tamponade, toxins and thrombo-embolism), however the difficulty for pre-hospital clinicians can be establishing if presentations such as hypothermia are the cause of a cardiac arrest, or a consequence of it.

This case report seeks to outline the case of an adult male patient who was rescued from a river and deteriorated into cardiac arrest. The report uses a structure adapted from the case report guidelines (CARE, 2013). He was hypothermic and underwent prolonged pre-hospital resuscitation, as well as transport to a receiving centre capable of providing extracorporeal life support (ECLS). ECLS is commonly known as a heart–lung bypass and allows a pump to take over the role of the heart and a membrane oxygenator that of the lungs, to exchange oxygen and carbon dioxide (Extracorporeal Life Support Organisation, 2022). The patient survived to hospital discharge with an excellent cerebral recovery.

## Case presentation

A 999 emergency call was received during the early hours of an April morning, to reports of an attempted rescue of a male from the river, by a bystander. At the time of this incident the patient was an unknown adult male, with an unknown past medical and medication history. The ambient air temperature recorded at the time of this incident was 4–6°C (World Weather Online, 2022). In addition to an ambulance crew, the hazardous area response team, enhanced care team (ECT, doctor-led asset) and police and fire services were also dispatched. The fire service launched a rescue boat to retrieve the patient from a riverbank to an area from which he could be extricated to an ambulance; he was in the water for approximately 45 minutes. At first contact with the fire service, the patient was reported to be shivering and conscious but confused. The patient was rescued to the riverbank for assessment by the attending ambulance crew, where he was found to have a Glasgow Coma Scale (GCS) score of 7/15 (eyes 4, voice 2, motor 1). The patient was moved to the heated ambulance saloon where all of his wet clothes were removed and he was covered with blankets. A baseline set of observations was obtained (see Table 1), however heart rhythm analysis was difficult due to shivering. There were no obvious injuries on initial assessment.

**Table 1.** Vital signs.

Respiratory rate	Heart rate	Blood pressure	Oxygen saturation	GCS score	Pupils	Blood glucose	Temperature
12	105	Not recorded	83% air	7/15	6 mm, reactive	11.2 mmol/L	LOW^a^

^a^Tympanic thermometers lowest temperature reading 34°C (Braun, 2014).

GCS: Glasgow Coma Scale.

Shivering made heart rhythm analysis difficult, given the artefact that was generated, although the patient did have a palpable carotid pulse on initial assessment. While obtaining a baseline set of observations, the patient was seen to take a deep breath, arch his back and deteriorate into cardiac arrest, 11 minutes after he was rescued from the water. Recorded heart rate was higher than expected and it is unclear if this is artefactual from data automatically transmitted to the patient record based on electrocardiogram information, or if this was manually recorded by the attending crew in this patient’s case.

### Initial presenting rhythm: pulseless electrical activity

The patient was initially managed as per the advanced life support algorithm with external chest compressions, assisted ventilation and 1 mg in 10 ml (1:10,000) intravenous adrenaline. Given the initial tympanic temperature reading of 34°C or below, the interval between doses of adrenaline was doubled to at least six to 10 minutes. At the point an oesophageal temperature probe was placed, and the core temperature recorded at 24°C, no further drugs were administered. 1000 ml of 0.9% sodium chloride was administered intravenously via a fluid warmer, while cardiopulmonary resuscitation was ongoing. Ventricular fibrillation (VF) was observed during rhythm assessments and defibrillation was administered on three occasions, but further attempts were discontinued given the core temperature was less than 30°C. The patient remained in VF for the remainder of the pre-hospital clinical encounter.

As the patient was seen to be alive immediately prior to the ECT arrival, and an oesophageal temperature of 24°C had been recorded, a decision was made to convey the patient to a hospital capable of delivering ECLS. This decision was balanced on the availability of a local emergency department within 10 minutes’ travel time and the ECLS centre involving a travel time of 45 minutes. Pre-hospital resuscitation was performed for more than 60 minutes.

A pre-alert was passed to the receiving emergency department, and on arrival the patient remained in cardiac arrest, with ongoing resuscitation. The patient was received in the emergency department by a resuscitation team, including the cardiothoracic consultant on call for the evening. A rapid handover was completed, and a mechanical chest compression device was applied (AutoPulse^®^). Once these actions were completed, the patient was moved immediately to theatre, in order for ECLS to be initiated.

## Follow-up and outcomes

The patient was established on femoral arterio-venous cardiopulmonary bypass and was rewarmed from 24°C. He spontaneously cardioverted from VF at 29°C.

Following rewarming and a short stay in intensive care, sedation was discontinued and the patient was successfully extubated with a GCS score of 15/15. Unfortunately, the patient had developed neurological weakness in his lower limbs due to a hypoxic spinal cord injury. Xie et al. (2017) note that the incidence of neurological complications is higher in patients undergoing ECLS for cardiac arrest or shock, but assert that it is unclear if these are due to the instigation of ECLS or the initial underlying pathology. The patient has subsequently been discharged home following a period of rehabilitation at a specialist spinal injury unit.

## Discussion

This case report highlights the positive impact that early recognition and management of cardiac arrest can have on the survival of patients (European Resuscitation Council, 2021). Although hypothermic cardiac arrest is an uncommon presentation given the temperate climate of the United Kingdom, there have been case reports of patients surviving from unintentional hypothermia (Coleman et al., 2010; Gani et al., 2016; Ko et al., 2002; Spooner & Hassani, 2000).

Data from the international hypothermia registry (established in 2010 at the University Hospital of Geneva) found that 48/73 hypothermic patients suffering cardiac arrest involved alpine incidents related to avalanche and crevasses, with 15% (11/73) attributed to water (Walpoth et al., 2021). VF was the presenting rhythm in 17 cases, asystole in 24 cases and pulseless electrical activity in six cases – further analysis of those related to water incidents was not possible. Twenty-six of the 73 patients survived to hospital discharge, which is comparable to other studies citing survival rates of 40.3% (Saczkowski, 2018).

Hypothermia is defined as a core body temperature of less than 35°C, and has previously been quantified using the Swiss hypothermia classification system, with an increased stage of hypothermia corresponding to a falling core temperature, loss of shivering and reducing conscious level (Musi et al., 2021). This system has been revised (see Table 2), given that the presence/absence of shivering was believed to be unreliable and other clinical findings correlated poorly with actual core body temperature. The new system focuses on identifying patients at increased risk of cardiac arrest, rather than the actual body temperature, using simplified assessment tools including the AVPU (alert, voice, pain, unresponsive) scale.

**Table 2.** Revised Swiss System for staging of accidental hypothermia.

	Stage 1	Stage 2	Stage 3	Stage 4
**Clinical findings**	‘Alert’ from AVPU	‘Verbal’ from AVPU	‘Painful’ or ‘Unconscious’ from AVPU **and** Vital signs present	‘Unconscious’ from AVPU **AND** No detectable vital signs
**Risk of cardiac arrest**	Low	Moderate	High	Hypothermic cardiac arrest

Note: Table adapted from Musi et al. (2021).

AVPU: alert, voice, pain, unresponsive.

The problem in the pre-hospital setting is that the widely used tympanic thermometers do not record sufficiently low enough to obtain meaningful values (e.g. the Braun ThermoScan 7 IRT 6520 recording range is 34–42.2°C ± 0.3°C below 35°C (Braun, 2014)). Alternative methods of recording temperature exist, such as rectal or oesophageal probes, but these are also not commonly used in UK ambulance services. These invasive probes are often impractical as they involve exposing the patient or cannot be used unless the patient is sedated or in cardiac arrest.

Identification of a core body temperature of less than 30°C allowed on-scene clinicians to amend their clinical management as per the Resuscitation Council UK (2021c) special circumstances guidelines as follows:

1. If VF persists after three shocks, delay further attempts until the core temperature is > 30°C.
2. Withhold adrenaline if the core temperature is < 30°C.
3. Hypothermic cardiac arrest rewarming should be performed with ECLS.
4. Non-ECLS rewarming should be initiated in a peripheral hospital if an ECLS centre cannot be reached within hours (e.g. 6 h).

In addition to the Resuscitation Council UK guidelines, the Joint Royal Colleges Ambulance Liaison Committee (2021) advises clinicians to withhold all advanced life support drugs when the core temperature is below 30°C – including amiodarone.

Unfortunately, a mechanical chest compression device was not available at this incident, and manual chest compressions were performed by rotating three members of staff during transit to the ECLS capable centre. It is recognised that a chest compression device is recommended during prolonged resuscitation attempts, due to the risk of provider fatigue. ECLS centres not only have the capability of increasing the temperature in a controlled manner (when compared to non-invasive methods that are less easily controlled); they can also offer cardiovascular and respiratory support during the rewarming and weaning process if required.

## Conclusion

The unpredictable nature of pre-hospital care necessitates that clinicians can quickly assimilate information and develop management plans that are versatile. Knowledge of the services that are available in the geographical area within which clinicians work gave them the ability to succinctly and clearly communicate the situation to an appropriate facility, which was capable of delivering the ECLS level of care that was required.

This case highlights that clinicians must be astute to the fact that hypothermia can present in UK pre-hospital practice and be so severe that it can lead to cardiac arrest, even in the spring months. Having an awareness of the ECLS-capable centres in the local area will help to minimise on-scene delays, should clinicians encounter a patient deemed suitable for this clinical intervention. Moving forward, this highlights the need for ambulance services to engage with the wider hospital network, to establish clear referral pathways for this niche patient group, as well as considering what additional equipment may be required, such as low-reading thermometers and mechanical chest compression devices.

### Patient perspective

The patient provided written consent for publication of this anonymised case report, and provided the following comment:

I’m just grateful for what you guys did and for not giving up on me. If my understanding is correct, you guys went past the point of calling it and as a result I am still here. The NHS is a great institution, underfunded, understaffed and over-worked. Yet still they go above and beyond and show that hard work, fast action and determination can save lives. It’s my hope that others learn from what happened to me and more lives are saved. I will always be grateful and sing your praises.

## Author contributions

SE acts as the guarantor for this article.

## Conflict of interest

None declared.

## Funding

None.
